# Does use of nonsteroidal anti-inflammatory drugs increase patients’ clinical severity of COVID-19?

**DOI:** 10.1590/1516-3180.2021.0401.27052021

**Published:** 2021-08-02

**Authors:** Henrique Souza Barros de Oliveira

**Affiliations:** I MD. Physician, Department of Medicine, Centro Universitário São Camilo (CUSC), São Paulo (SP), Brazil

Dear Editor,

Since the emergence of the first cases of coronavirus disease 2019 (COVID-19) at the end of 2019, understanding of this disease has been advancing rapidly. Updates on this subject are crucial to healthcare professionals who are engaged in the battle against this disease. However, it is noteworthy that the emergence of updates not based on evidence has generated difficulties in the clinical management of patients in the midst of this pandemic, especially when dealing with cases of misleading information.

In March 2020, the French Minister of Health (Oliver Veran) used the social network Twitter to announce that unpublished data indicated that use of nonsteroidal anti-inflammatory drugs (NSAIDs) could increase the severity of COVID-19, possibly due to viral replication mechanisms. This comment seems to have originated in part from the clinical opinion of an infectious disease specialist in southwestern France, where it was observed that four cases of previously healthy young patients with COVID-19 progressed to the severe stage of the disease after previous use of NSAIDs to combat the initial phase symptoms. This announcement had worldwide repercussions and led to clinical insecurity regarding drug therapy for patients with COVID-19, despite the lack of any scientific evidence.^1^^,^^2^

Given this scenario, regulatory authorities have tried to investigate the association between use of NSAIDs, especially ibuprofen, and patients’ severity of COVID-19.^1^^,^^2^ Some studies have been conducted in different populations, but failed to show any significant association between use of NSAIDs and increased risk of severe clinical disease among patients with COVID-19.^1^^,^^3^^,^^4^ Among these, Drake et al. developed a prospective cohort study between 17 January and 10 August 2020, on 78,674 hospitalized patients with a confirmed diagnosis of COVID-19 at 255 healthcare services in England, Scotland and Wales. After adjusting the sample, it was noted that use of NSAIDs was not associated with worsened mortality (odds ratio, OR 0.95; 95% confidence interval, CI 0.84-1.07; P = 0.35), starting to need intensive care (OR 1.01; 95% CI 0.87-1.17; P = 0.89), requirement of oxygen therapy (OR 1.0; 95% CI 0.89-1.12; P = 0.97), invasive ventilation (OR 0.96; 95% CI 0.80-1.17; P = 0.69), non-invasive ventilation (OR 1.12; 95% CI 0.96-1.32; P = 0.14) or occurrence of acute renal injury (OR 1.08; 95% CI 0.92-1.26; P = 0.33). However, despite the limitations of that study, it should also be noted that rational use was made of this pharmacological group, especially among elderly individuals and patients with multimorbidity.^1^

NSAIDs are considered to be potentially inappropriate medicines (PIMs) for use among the elderly, due to these individuals’ senescence. They should be prescribed with caution, given that the risk might be greater than the clinical benefits provided, while safer and more effective alternatives may be available. Thus, they can be considered potentially inappropriate.^5^

Consumption of NSAIDs is commonplace, especially in age groups ≥ 60 years, since they are dispensed readily in pharmacies in Brazil, without any requirement to restrict medical prescriptions. This access to medications that are considered to be over-the-counter (OTC) drugs by Brazil’s national regulatory agency facilitates occurrence of self-medication, polypharmacy, drug interactions and imminent risk of adverse reactions. However, it has been recognized that use of NSAIDs potentially gives rise to health risks, through promoting salt and water retention, reducing inhibition of chloride absorption and reducing the action of the antidiuretic hormone, thus facilitating a propensity to hyperkalemia, edema and decompensation of blood pressure levels, especially in hypertensive individuals. In addition, there are risks of dyspepsia, heartburn, gastric and duodenal ulceration, increased risk of bleeding time due to disorders of platelet function, acute renal injury due to renal hypoflux and decreased glomerular filtration rate.^5^

In view of the above, it is noteworthy that, to date, there has been no significant evidence for any increase in clinical severity of COVID-19 among patients who use of NSAIDs, especially ibuprofen. Nonetheless, this scenario brings a need to review the form of prescription among patients with COVID-19 and the form of dispensing this pharmacological group.
